# Fruit and vegetable consumption among people living with HIV at a large Urban HIV Clinic in Central Uganda: A cross-sectional study

**DOI:** 10.1371/journal.pone.0361755

**Published:** 2026-11-06

**Authors:** Martin Kasendwa, Ritah Nantale, Ronald Kusolo, Roy William Mayega, Rawlance Ndejjo, Geofrey Musinguzi, David Mukunya, Aloysius Ssennyonjo, Daniel Murokora, Ahmed Ddungu, Noela Clara Owarwo, Norah Jackie Nanteza, Barbara Asire, Rhoda Wanyenze

**Affiliations:** 1 Department of Disease Control and Environmental Health, School of Public Health, Makerere University, Kampala, Uganda; 2 Department of Community and Public Health, Faculty of Health Sciences, Busitema University, Mbale, Uganda; 3 Babies and Mothers Alive Foundation, Kampala, Uganda; 4 Infectious Diseases Institute, Makerere University College of Health Sciences, Kampala, Uganda; 5 Seed Global Health, Kampala, Uganda; 6 United Nations Children's Fund (UNICEF), Kampala, Uganda; Fayetteville State University, UNITED STATES OF AMERICA

## Abstract

**Background:**

Fruit and vegetable consumption has been shown to effectively reduce the risk of developing non-communicable diseases such as hypertension. This study assessed the prevalence, perceptions and factors associated with fruit and vegetable consumption among people living with HIV (PLHIV) at an Urban HIV clinic in central Uganda.

**Methods:**

This was a cross-sectional study conducted at the Infectious Diseases Institute (IDI) HIV clinic located in Kampala, Uganda. An adapted World Health Organisation STEPwise questionnaire was administered with Open Data Kit (ODK) to a random sample of 431 PLHIV clients aged 18 years and older to obtain data on levels and perceptions of fruit and vegetable consumption. Adequate intake of fruits and vegetables was defined as consumption of five or more servings of fruits and/ or vegetables per day in a typical week. We conducted multivariable robust Poisson regression to determine the independent factors associated with adequate fruit and vegetable consumption. Data were analyzed in Stata version 17.0.

**Results:**

A total of 44 out of 417 (10.6%) respondents met the WHO recommendations of fruit and vegetable consumption. Overall, the perceptions of respondents about consumption of a fruits and vegetables were favourable (mean = 3.7, SD = 0.5), with the construct of perceived barriers to eating fruits and vegetables (mean = 3.2, SD = 1.6) having the least favourable perceptions. Being unemployed (APR: 0.1, 95% CI:0.0–0.8, p = 0.027), having a higher household income (APR:0.99, 95% CI: 0.900–0.998, p = 0.039) and having a longer duration on Antiretroviral Therapy (ART) (APR:0.9, 95% CI:0.89–0.996, p = 0.046) were significantly associated with inadequate consumption of fruits and vegetables.

**Conclusions:**

Fruit and vegetable consumption among PLHIV was low. Our findings underscore the need for targeted strategies and interventions to promote healthy dietary practices, reduce perceived barriers, and improve awareness of hypertension risk among PLHIV.

## Background

Hypertension among people living with HIV (PLHIV) is a major public health concern, with a global prevalence estimated at 25.2% [1]. In sub-Saharan Africa, approximately 21.9% of PLHIV have hypertension [2]. Studies conducted in West, East, and Southern Africa have reported a high burden of hypertension among PLHIV, with prevalence rates ranging from 15.1% to 50.1% [3–7]. In Uganda, studies conducted among PLHIV receiving care in urban HIV treatment centers have reported hypertension prevalence rates ranging from 35.2% to 58.2% [4,8,9]. The increasing prevalence of hypertension among PLHIV is linked to a combination of risk factors, including advancing age, the direct effects of HIV infection, certain antiretroviral therapy (ART) regimens, and unhealthy lifestyle behaviors. [10,11].

Fruit and vegetable consumption has been shown to effectively reduce the risk of developing non-communicable diseases, including hypertension [12]. PLHIV often have compromised immune systems, leading to inflammation and an increased risk of chronic diseases and infections [13,14]. As such, they require a balanced intake of fruits and vegetables, which provide essential micro- and macronutrients to support immune function and alleviate symptoms [13,14]. Potassium found in fruits and vegetables has been shown to lower blood pressure [15,16]. In addition, dietary fibre may contribute to blood pressure reduction and, along with phytochemicals such as plant sterols, flavonoids, and other antioxidants, play a vital role in regulating cholesterol levels and other biological processes [16,17].

Studies have investigated dietary practices among PLHIV in Africa, highlighting unhealthy dietary patterns in this population with a predominance of starchy staples. A recent study among PLHIV accessing care at Narok County Referral Hospital in Southwestern Kenya found that carbohydrates were being consumed in excess of the recommended amounts, whereas the consumption of fruits and vegetables was low [18]. Furthermore, a study conducted in Uganda among adults, revealed that only 12.2% of them consumed five or more servings of fruits and/or vegetables per day in a typical week [19]. Several factors including employment status, WHO HIV clinical stage, age, marital status, and household income have been associated with consumption of fruits and vegetables among PLHIV [14,18,19].

The World Health Organization (WHO) recommends a daily intake of 400 g of fruits and vegetables, equivalent to five portions, to mitigate the risk of chronic diseases [20]. In Uganda, there is limited data on fruit and vegetable consumption among PLHIV. This study assessed the prevalence, perceptions and factors associated with fruit and vegetable consumption among PLHIV receiving care at an urban HIV clinic in Uganda.

## Methods

### Study design

This was a cross-sectional study employing quantitative methods of data collection, conducted between 20/07/2023 and 30/08/2023 using an Open Data Kit (ODK) adopted from the World Health Organization's STEPwise questionnaire to assess the prevalence and perceptions regarding fruit and vegetable consumption among people living with HIV (PLHIV).

### Study setting

This study was conducted at the Infectious Diseases Institute (IDI), a large private not-for-profit multi-disciplinary HIV clinic under Makerere University’s stewardship located within Mulago National Referral Hospital complex in Kampala, Uganda. The clinic, established in 2002, is a center of excellence that provides free HIV care and treatment to over 8,400 patients with funding from the U.S. President’s Emergency Plan for AIDS Relief (PEPFAR). The clients in care are enrolled into differentiated service delivery models based on their preferences, duration on antiretroviral therapy (ART), and clinical stability, particularly with regard to HIV viral load suppression and co-existing conditions, such as hypertension. In 2014, the prevalence of hypertension among PLHIV at IDI was estimated at 15.1% [3] and this has since increased to about 24.4% according to a study published in 2023 [21]. Most patients served by the clinic reside in Kampala and Wakiso, which are predominantly urban and peri-urban districts undergoing rapid urbanization. As part of the routine care, the clinic provides regular screening, diagnosis, and management of NCDs, including hypertension. This also includes individualized lifestyle modification counselling aimed at improving long-term health outcomes for PLHIV.

### Study population

People living with HIV (PLHIV) aged 18 years and above receiving care at the Infectious Diseases Institute (IDI) HIV clinic. We included PLHIV aged 18–69 years who had spent at least 6 months on ART, scheduled to attend the HIV clinic during the study period, and able and willing to give written informed consent to participate in this study. Patients who were transferred in on ART with an undocumented ART start date and had been under care at the clinic for less than 6 months were excluded. We also excluded PLHIV who were ill, unable to communicate, or who had physical disabilities which would limit undertaking of physical activity recommendations.

### Sample size estimation

The sample size was calculated using Kish and Leslie formula [22] based on the following assumptions: a 5% level of significance, assumed prevalence of 50% for fruit and vegetable consumption (because no sufficiently comparable prevalence estimate was available at the time of study planning), an error margin of 5% and a 10% non-response rate. This gave us a minimum sample size of 427 participants.

### Sampling procedure

On a daily basis, the Data Manager was requested for a list of clients who were scheduled for appointment, from which the list of clients that met the inclusion/ exclusion criteria was obtained. From each daily list, 60 scheduled clients meeting the study criteria were randomly selected without replacement until the sample size was attained. On days when the scheduled clients meeting the inclusion/ exclusion criteria were less than 60, all the clients were selected to participate in the study. On the final data collection day, the number of clients needed to meet the required sample size was less than the daily target of 60 and therefore, the pending number of clients was sampled.

### Study variables

Our primary outcome variable was adequate fruit and vegetable consumption. Adequate intake of fruits and vegetables was defined as consumption of five or more servings of fruits and/ or vegetables per day in a typical week, in line with the recommendations by the World Health Organization [20].

The independent variables included;

Demographic factors such as age, sex, marital status, and level of education, and Body Mass Index (BMI). BMI was classified according to the WHO as underweight ((< 18.5 kg/m2), normal weight (18.5–24.9 kg/m2), overweight (25–29.9 kg/m2), and obesity (≥ 30 kg/m2) (WHO, 2000).

Social economic factors comprised annual household income, and employment status.

HIV disease-related factors such as duration on ART, WHO HIV clinical stage, Viral Load Status, and ART regimen category/ line of treatment. Viral load was defined as suppressed if the most recent result documented in the client’s file was less than 1000 copies of HIV RNA per milliliter of blood, and non-suppressed if ≥1000 copies of HIV RNA per millilitre of blood.

History of at least one of the following conditions: 1) hypertension, 2) diabetes, 3) cardiovascular disease, 4) raised total cholesterol.

Perceptions of fruits and vegetables consumption for the prevention and control of hypertension: A set of questions derived from review of literature [23,24] were considered under five constructs of the Health Belief Model (HBM). These included perceived susceptibility to hypertension, perceived severity of hypertension, perceived benefits of fruits and vegetables consumption, perceived barriers to consuming fruits and vegetables, and perceived self-efficacy for fruits and vegetables consumption. The responses to each question under each construct were captured using a 5-point Likert scale ranging from strongly agree (code 1), agree (code 2), neutral (code 3), disagree (code 4) and strongly disagree (code 5). The responses to each question on perceptions were further categorized in two broad categories of “Agree” (for strongly agree and agree), and “Disagree” (for neutral, strongly disagree, and disagree) and the frequency of these two categories obtained and reported. An average perception score was ascertained for each question. This was followed by computation of construct-specific scores by obtaining the average of perception score for the underlying questions. For each construct, overall perceptions were considered to be favourable if the computed average score for all the perception questions was equal or above 3, and unfavorable if it was less than 3.

### Data collection and quality control

Data collection was done by a team of eight trained research assistants with experience in conducting patient-level interviews in a very busy setting. A pilot test was conducted at Kawaala health centre IV ART clinic, which was outside the study site. The expert clients at the HIV clinic sensitized the selected participants about the study and study procedures. This was done as the clients exited the clinic to ensure that the study does not unnecessarily increase their waiting time and or disrupt the normal client flow. The selected participants were directed to research assistants for consenting and data collection/ interviewing. HIV disease related data including the most recent viral load, WHO clinical stage, duration on ART, and ART regimen category for the interviewed clients were abstracted from the electronic medical records. These procedures were repeated daily until the required sample size was obtained. The site Clinic Manager and the principal investigator further provided day-to-day supervision of the research assistants during the data collection exercise.

### Data management

The survey tool was programmed in an android based open data kit (ODK) platform. In-built data validations were used to ensure data quality through skip patterns, logic checks for erroneous data and completeness. Data were uploaded daily to a secure ODK central server by the research assistants. The data manager downloaded the data on a daily basis to review it for completeness and consistency. In situations of inconsistencies or incompleteness, the field teams were contacted to review the facility charts to retrieve the missing information.

### Statistical analysis

We summarized participant characteristics using frequencies and percentages for categorical variables and by means (standard deviation) or medians (interquartile range) for continuous variables as appropriate. The proportion of individuals meeting the recommended servings of fruits, servings of vegetables and servings of fruits or vegetables were also computed.

We determined the factors associated with adequate fruits and vegetables consumption using the modified Poisson model with robust standard errors to obtain prevalence ratios (PR) and their corresponding 95% confidence intervals. Variables with a p  <  0.2 at bivariable level, those known to be associated with physical activity from literature and those considered plausible although not significant, were entered into the multivariable model to determine the independent factors associated with adequate fruits and vegetables consumption. Statistical significance was set at a p-value less than 0.05. For clinically abstracted variables, including viral load, analyses were conducted using available data, and participants with missing values for specific variables were excluded from analyses involving those variables only. Data were analysed in Stata version 17.0

### Ethical considerations

This study was conducted in accordance with the Declaration of Helsinki. The study was approved by Makerere University School of Public Health Research and Ethical Committee, located at the School of Public Health, Makerere University, which is an accredited Institutional Review Board (IRB), approval (number MakSPH-REC-2023–229) and administrative clearance from the Infectious Disease Institute (IDI) administration. We sought written informed consent from all study participants before enrollment into the study. Participation in the study was voluntary, and participants had the right to withdraw at any time.

Privacy and confidentiality of data were ensured by masking participants with personal identifiers like name, and was addressed by assigning a unique code to each participant. Participants were free to accept or refuse to participate in the study.

## Results

### Participant characteristics

A total of 431 participants were recruited in this study. More than a half (65%) were female, and were aged 30–49 years (55.7%). Most participants had completed primary school education (36.2%), were employed (80.3%), and were either currently married or cohabiting (52.4%). Over half (58.2%) did not report any history of raised blood pressure, diabetes mellitus, raised total cholesterol, or cardiovascular diseases (CVD). Most (47.8%, 192/402) had a normal BMI (18.5–24.9 kg/m2), and 29 participants were pregnant hence their BMI was not calculated. The median monthly household income was 71 USD (IQR: 28–143 USD). Clinically, most of the participants were in WHO HIV clinical stages 3 and 4 (32.6%, 138/424; 27.4%, 116/424 respectively), had a suppressed HIV RNA viral load (97.9%, 417/426), and were on first line ART regimens (74.5%, 321/431). The median duration on ART was 11 years (IQR: 7.6–15.3 years). Details are in Table 1.

**Table 1:** Characteristics of the study participants.

Variable	Frequency (n)	Percentage %
**Sex (n=431)**		
Male	151	35.0
Female	280	65.0
**Age in years (n=431)**		
18-29	40	9.3
30-49	240	55.7
50-69 years	151	35.0
**Level of education attained (n=431)**		
No formal schooling	99	23.0
Primary School completed	156	36.2
Secondary School Completed	122	28.3
Tertiary Education	54	12.5
**Marital status (n=431)**		
Never married	58	13.5
Currently married/Cohabiting	226	52.4
Separated/Widowed/Divorced	145	33.6
Not stated	2	0.5
**Employment Status (n=431)**		
Employed	346	80.3
Unemployed	85	19.7
**WHO HIV Clinical Stage (n=424)**		
Stage 1	69	16.3
Stage 2	101	23.8
Stage 3	116	27.4
Stage 4	138	32.6
**Viral Load Status (n=426)**		
Suppressed	417	97.9
Non-suppressed	9	2.1
**ART Regimen Category (431)**		
First Line	321	74.5
Second Line	92	21.4
Third Line/ Complex	18	4.2
**Body Mass Index (kg/m^2^) (n=402)**		
Under Weight, < 18.5	41	10.2
Normal Weight, 18.5–24.9	192	47.8
Overweight, ≥ 25.0 – 29.9	91	22.6
Obesity, ≥ 30	78	19.4
**History of Raised Blood Pressure or Diabetes or raised total cholesterol or CVD (n=431)**		
Yes	180	41.6
No	251	58.2
**Variable**	**Median**	**IQR**
**Median monthly household income (UGX) (n=431)**	71	28-143
**Median duration on ART (In years) (n=431)**	28-143	7.6 -15.3

### Levels of fruit and vegetable consumption among people living with HIV in Uganda

A total of 417 (96.8%) respondents reported consumption of fruits and/or vegetables, 10.6% (44/417) met the WHO recommendation of consuming 5 or more servings of fruits and/or vegetables per day in a typical week. More respondents met the WHO recommendation through consumption of fruits alone (8.6%, 35/406) compared to vegetables alone (3%, 12/406) (Fig 1).

**Fig 1:**
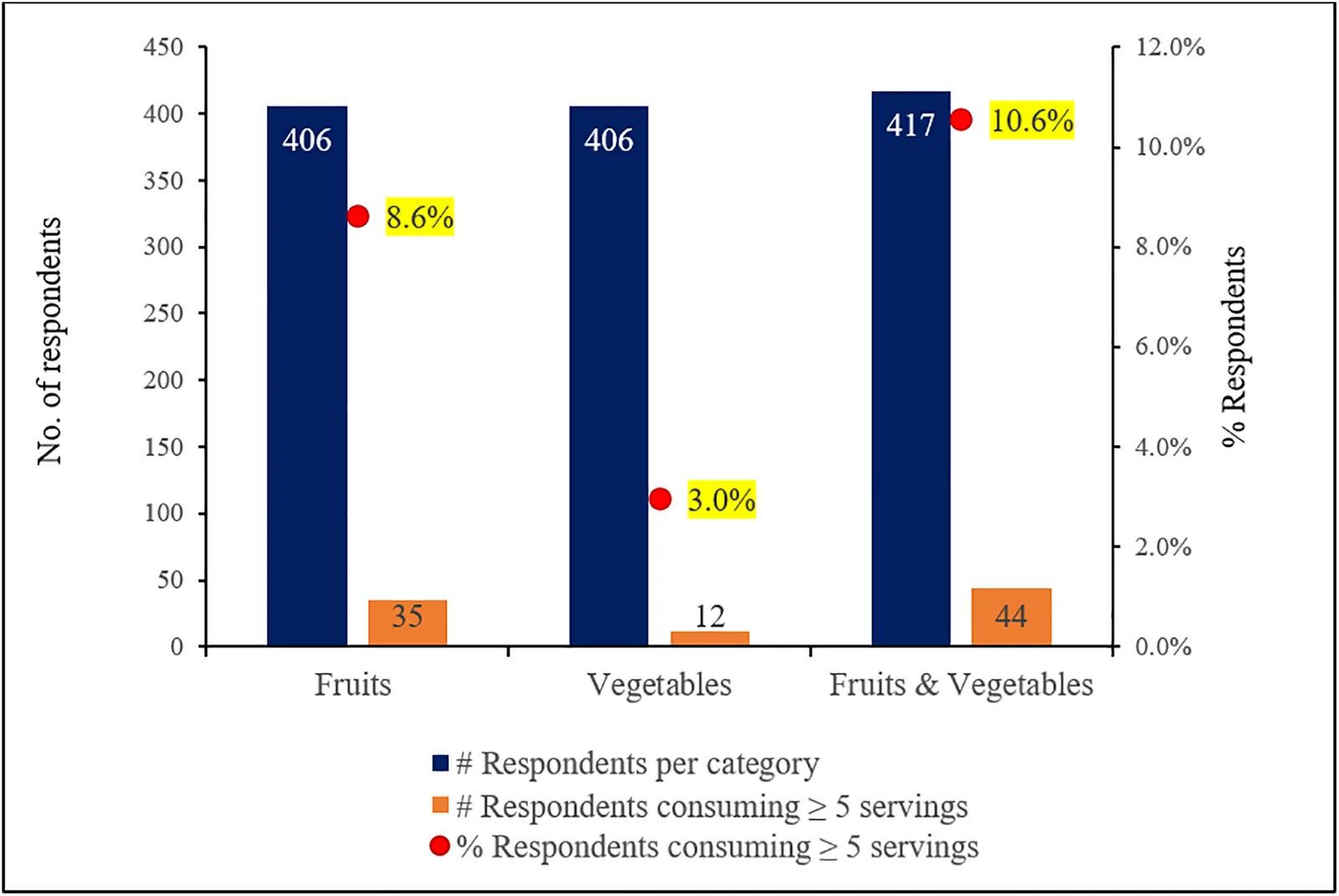
A clustered column chart showing the percentage of respondents consuming the recommended servings of fruit and/or vegetables per day in a typical week.

### Perceptions of people living with HIV regarding fruit and vegetable consumption for the control of hypertension in Uganda

Most of the participants (86.1%, 371/431) perceived high blood pressure to be a serious health threat but only 57.3% (247/431) felt susceptible to the condition. Majority (82.1%, 354/431) of the respondents perceived that eating fruits and vegetables could help them to prevent or control hypertension. However, most participants perceived the high cost (47.3%, 204/431) and limited variety (42.0%, 181/431) of fruits and vegetables at eateries as major barriers to their consumption. Regarding self-efficacy for fruits and vegetables consumption, 81.2% (350/431) respondents were confident that they could consume more fruits and vegetables regularly. Advice from a health worker was the most prevalent cue to adoption of a healthy diet (96.5%, 416/431), followed by encouragement from friends and family members (79.4%, 342/431), and social media reminders (58.7%, 253/431). Overall, most of the participants had favourable perceptions (average scores ≥3 on a scale of 1–5) for each of the 5 constructs of the HBM. The constructs with the most favourable perceptions were perceived severity of hypertension (mean = 4.2, SD = 0.9), and perceived benefits of fruits and vegetables consumption (mean = 4.0, SD = 0.8) (Table 2).

**Table 2:** Perceptions of people living with HIV regarding fruit and vegetable consumption for the control of hypertension in Uganda.

Perceptions	Agree n (%)	Disagree n (%)	Mean (SD)
**Perceived susceptibility to hypertension**			3.2 (1.6)
My chances of getting hypertension are high	247 (57.3)	184 (42.7)	
**Perceived severity of hypertension**			4.2 (0.9)
It is serious to have high blood pressure.	371 (86.1)	60 (13.9)	
**Perceived benefits of fruits and vegetables consumption**			4.0 (0.8)
Eating fruits and vegetables can help me to prevent or control hypertension.	354 (82.1)	77 (17.9)	
**Perceived barriers to fruits and vegetables consumption**			3.5 (0.6)
Too expensive	204 (47.3)	227 (52.7)	
Limited choice of fruits and vegetables at eateries	181 (42.0)	250 (58.0)	
Fruits and vegetables do not taste good	35 (8.1)	396 (91.9)	
Family members do not like fruits and vegetables	40 (9.3)	391 (90.7)	
**Perceived self-efficacy for fruits and vegetables consumption**			3.8 (1.0)
Confident in my ability to consume more fruits and vegetables regularly.	350 (81.2)	81 (18.8)	
**Overall perceptions about fruits and vegetables consumption**	NA	NA	3.7 (0.5)
**Cues to action for fruits and vegetables consumption**			
Advice from a health worker	416 (96.5)	15 (3.5)	
Encouragement from friends & family members.	342 (79.4)	89 (20.6)	
Social media reminders.	253 (58.7)	178 (41.3)	

NA: frequency data are not applicable for this composite measure.

### Factors associated with adequate fruit and vegetable consumption among people living with HIV in Uganda

Employment status (APR:0.1, 95% CI:0.0–0.8, p = 0.027), average household monthly income (APR:1.0, 95% CI:0.9–1.0, p = 0.039), and duration on ART (APR:0.9, 95% CI:0.9–1.0, p = 0.046) were found to be significantly associated with meeting the WHO recommended servings of fruits and vegetables (Table 3).

**Table 3:** Factors associated with adequate fruit and vegetable consumption among people living with HIV in Uganda.

Characteristic	# of servings ≥5 (%)	Crude PR [95% CI]	p-value	Adjusted PR [95% CI]	p-value
**Sex**					
Male	21 (14.4)	1.0		1.0	
Female	23 (8.5)	0.6 (0.3-1.0)	0.063	0.8 (0.4-1.6)	0.485
**Age in years**					
18-29 years	2 (5.0)	1.0		1.0	
30-49 years	22 (9.6)	1.9 (0.5-7.8)	0.367	1.2 (0.3 - 4.8)	0.777
50-69 years	20 (13.6)	2.7(0.7-11.2)	0.165	2.2 (0.5–9.2)	0.295
**Education Level**					
No formal education	13 (13.8)	1.0		1.0	
Primary School	14 (9.3)	0.7 (0.3-1.4)	0.278	0.7 (0.4-1.5)	0.373
Secondary School	10 (8.3)	0.6 (0.3-1.3)	0.203	0.8 (0.4-1.7)	0.526
Tertiary School	7 (13.2)	1.0 (0.4-2.2)	0.916	1.4 (0.6-3.4)	0.410
**Marital Status**					
Married/Cohabitig	26 (11.9)	1.0		1.0	
Unmarried (Never married /Separated/ Widowed/ Divorced)	18 (13.0)	0.8 (0.4-1.4)	0.377	0.8 (0.4-1.6)	0.544
**Employment Status**					
Employed	42 (12.5)	1.0		1.0	
Unemployed	2 (2.4)	0.2 (0.1-0.8)	0.377	0.1 (0.0–0.8)	0.027*
**WHO HIV Clinical Stage**					
Stage 1	3 (4.4)	1.0			
Stage 2	12 (12.4)	2.8 (0.8–9.7)	0.095	2.7 (0.8–9.3)	0.122
Stage 3	13 (11.9)	2.7 (0.8–9.3)	0.105	2.7 (0.8–9.6)	0.108
Stage 4	15 (11.1)	2.6 (0.8–8.5)	0.125	2.5 (0.7–8.6)	0.145
**Viral Load Status**					
Suppressed	43 (10.6)	0.9 (0.1–5.5)	0.865	0.7 (0.1–5.8)	0.717
Non-suppressed	1 (12.5)	1.0		1.0	
**ART Regimen category**					
First Line	34 (10.9)	1.0		1.0	
Second Line	7 (8.0)	0.7 (0.3–1.6)	0.424	0.9 (0.4–1.9)	0.719
Third Line/ Complex	3 (16.7)	1.5 (0.5–4.5)	0.445	1.6 (0.5–5.6)	0.461
**History of diabetes or cholesterol or CVD**					
No	25 (10.4)	1.0		1.0	
Yes	19 (10.8)	1.0 (0.6-1.8)	0.890	0.9 (0.5-1.8)	0.854
**BMI**					
Normal weight	20 (10.9)	1.0		1.0	
Underweight	3 (7.7)	0.7 (0.2-2.3)	0.555	0.7 (0.2-2.3)	0.583
Overweight	10 (11.1)	1.0 (0.5-2.1)	0.964	1.0 (0.5-2.2)	0.847
Obesity	10 (13.2)	1.2 (0.6-2.5)	0.609	1.5 (0.7-3.0)	0.301
**Overall perceptions of fruits and vegetables consumption**					
Score of 1 to <3	3(9.4)	1.0		1.0	
Score of ≥3–5	41(10.7)	1.1 (0.4-3.5)	0.823	1.4(0.4-4.6)	0.579
**Average monthly household income**		0.9 (0.9-1.0)	0.082	0.99 (0.900-0.998)	0.039*
**Average duration on ART (years)**		1.0 (0.9–1.1)	0.350	0.9 (0.89–0.996)	0.046*

PR = Prevalence Rate, 95% CI = Confidence Interval, *Statistically Significant at P-value < 0.05.

## Discussion

In this facility-based cross-sectional study, we determined the prevalence, perceptions and factors associated with fruit and vegetable consumption among people living with HIV receiving care at an Urban HIV Clinic in Uganda. The prevalence of adequate fruit and vegetable consumption among the participants was low (10.6%). Being unemployed, having a higher household income and having a longer duration on Antiretroviral Therapy (ART) were significantly associated with adequate fruit and vegetable consumption.

Our study revealed inadequate intake of fruits and vegetables among PLHIV accessing care at the HIV clinic, with only 10.6% of participants meeting the recommended minimum of five or more servings of fruits and/ or vegetables per day in a typical week. This low consumption may be explained by several factors, including cultural dietary patterns characterized by a predominance of starchy staple foods, where fruits and vegetables are often considered accompaniments rather than essential components of meals. In addition, the ongoing nutrition transition, marked by increased consumption of Western-style diets rich in fats, refined sugars, and processed foods [25] may further contribute to suboptimal intake. Limited awareness of the health benefits associated with regular fruit and vegetable consumption may also play a role. These results corroborate findings of previous studies conducted among PLHIV in Uganda [26] and Kenya [18,27], and the general population in Uganda [19,28] and sub-Saharan Africa [29]. These findings highlight the need for targeted interventions, including nutrition education, mass awareness campaigns, and social and behaviour change communication strategies to promote healthier dietary practices among PLHIV. However, our findings differ from studies conducted in Ethiopia and Tanzania, which reported higher prevalence of adequate fruit and vegetable consumption among PLHIV [14,30]. These differences may reflect variations in study populations, dietary assessment methods, socioeconomic contexts, and availability and affordability of fruits and vegetables across settings.

Our study revealed that majority of the participants had favourable perceptions regarding the benefits of fruit and vegetable consumption, with 82.1% believing that it could help them to prevent or control hypertension. These results are in agreement with those obtained by Norsyahidah and colleagues among Malay adults, which highlighted prevention and management of diseases like high blood pressure among the perceived benefits of fruit and vegetable consumption [31].

Pertaining to barriers to fruit and vegetable consumption, our study highlighted the high cost of fruits and vegetables and the limited choice of fruits and vegetables at eateries as the most reported ones. These results reflect those of a recent qualitative study conducted in Kampala Metropolitan Area in Uganda which highlighted cost among the barriers [26]. This finding was also reported by a study done in Nigeria [32]. Similarly, most participants from a previous study in the United States of America reported that fast food places did offer enough choices of fruits and vegetables on their menus [33].

Regarding perceived self-efficacy for fruit and vegetable consumption, majority (81.2%) of the respondents in this study were confident that they could consume more fruits and vegetables regularly. This points to a high likelihood of consumption of fruits and vegetables by participants in our study based on positive associations between self-efficacy and fruit and vegetable consumption reported in earlier studies [34]. However, a previous study conducted in the United States of America reported a lower self-efficacy (54%) among adults to consume fruits and vegetables [33]. These variations could be related to other factors such as household income, cost of fruits and vegetables, availability of fruits and vegetables, and cultural differences.

We found a significant association between employment status and consumption of a healthy diet, with unemployed participants being less likely to consume the WHO recommended servings of fruits and /or vegetables per day in a typical week. This finding is consistent with findings from previous studies in Uganda [28] and South Africa [35]. This observation may be related to a lower likelihood of earning income and therefore a lower likelihood of affording fruits and vegetables by the unemployed compared to the employed. However, our finding was contrary to a study conducted in Ethiopia which reported a significant association between being unemployed and consuming the recommended amounts of fruits and vegetables [36]. This could be related to the tendency for fruits and vegetables to be less expensive compared to options such as animal proteins in some settings, which favours their selection.

We also found out that intake of adequate servings of fruits and vegetables was associated with average household income, with respondents from households with a higher monthly income earning being less likely to consume the WHO recommended servings of fruits and vegetables per day in a typical week. This was one of the striking outcomes, and a possible explanation for this observation could be an increasing tendency for households with higher average monthly incomes to gravitate towards Western diets despite the higher ability to afford fruits and vegetables accorded to them by the better financial status. However, other studies have obtained varying findings, a recent rapid review exploring factors affecting fruit and vegetable consumption and purchase behaviour of adults in sub-Saharan Africa reported household income among the most consistent factors positively associated with consumption of fruits and vegetables [37]. This could be related to cultural differences and varying preferences across contexts.

Regarding the association between HIV-related clinical factors and fruit and vegetable consumption, we found that PLHIV who had been on ART for longer durations were less likely to consume the WHO recommended servings of fruits and vegetables. This aligns with findings of three earlier studies conducted in Ethiopia which reported lower likelihood of dietary diversity among HIV patients who had been on ART for a shorter duration [38–40]. This could be attributed to greater adherence to health worker’s dietary guidance at the start of HIV care and treatment, and sliding back into old dietary habits and staple foods with increasing ART experience and improvement in HIV clinical status.

Our study had some limitations. First, we used the cross-sectional design which limits making any causal inferences for any associated explanatory variables and the outcome variables. Second, data on fruit and vegetable consumption were obtained through self-reporting. This could subject our results to recall bias and social desirability bias. The Health Belief Model constructs used in this study were adapted from previously validated measures; however, we did not assess their internal consistency using Cronbach’s alpha in the current study. Additionally, the categorization of perception scores into favourable and unfavourable categories was based on the direction of responses rather than a validated threshold, which may have reduced the variability of responses and introduced potential misclassification. Finally, our study was a single-center study and therefore our findings may not be generalizable to the broader population of PLHIV in Uganda.

## Conclusion

Fruit and vegetable consumption among PLHIV was low. Being unemployed, having a longer duration of ART, and a higher average monthly household income were significantly associated with inadequate intake. While overall perceptions of a healthy diet were positive, low perceived susceptibility to hypertension and high perceived barriers to healthy eating were the major hindrances to adequate fruit and vegetable consumption. Future research should evaluate the effectiveness of social and behaviour change communication approaches, to improve fruit and vegetable consumption among PLHIV.

## Supporting information

S1 File Fruit and vegetable consumption among PLHIV data. (XLSX)
