# Supplementary material for: Fruit and vegetable consumption among people living with HIV at a large Urban HIV Clinic in Central Uganda: A cross-sectional study
Source: PLoS One. 2026 Nov 6;21(11):e0361755. doi: 10.1371/journal.pone.0361755 (PMC13742917; doi:10.1371/journal.pone.0361755)
Supplement: Supplementary file 1 [file pone.0361755.s003.docx]

**Re: “PONE-D-26-17920**

**Fruit and vegetable consumption among people living with HIV at a large Urban HIV Clinic in Central Uganda: a cross-sectional study”**

Thank you for reviewing our manuscript.

Below is our point-by-point response to each comment.

Thank you.

| **Comment** | **Response to comment** | **Line number** |
| --- | --- | --- |
| **Editor** |  |  |
| 1. Please ensure that your manuscript meets PLOS ONE's style requirements, including those for file naming. | Thank you, this has been done. | NA |
| 2. We note that you had received approval to conduct research from Uganda National Council of Science and Technology (UNCST) number HS3597ES. If this is the case, please add this information to your manuscript and specify that this institution granted permission to conduct research. | We received from approval from Makerere University School of Public Health Research and Ethical Committee, approval (number MakSPH-REC-2023-229) and we highlighted this in the manuscript under Ethics approval and consent to participate. | 354 |
| 3. Please include your full ethics statement in the ‘Methods’ section of your manuscript file. In your statement, please include the full name of the IRB or ethics committee who approved or waived your study, as well as whether or not you obtained informed written or verbal consent. If consent was waived for your study, please include this information in your statement as well. | Thank you, this has been done. | 354-361 |
| 4. Please provide a complete Data Availability Statement in the submission form, ensuring you include all necessary access information or a reason for why you are unable to make your data freely accessible. If your research concerns only data provided within your submission, please write "All data are in the manuscript and/or supporting information files" as your Data Availability Statement. | Thank you, this has been done. | 368 |
| 5. Please amend your list of authors on the manuscript to ensure that each author is linked to an affiliation. Authors’ affiliations should reflect the institution where the work was done (if authors moved subsequently, you can also list the new affiliation stating “current affiliation:….” as necessary). | Thank you, each author is linked to an affiliation. | 4-5 |
| 6. We note you have included a table to which you do not refer in the text of your manuscript. Please ensure that you refer to Table 1, 2, 3, in your text; if accepted, production will need this reference to link the reader to the Table. | Thank you, this has been done. | 199,226,236 |
| 7. [Please include captions for your Supporting Information files at the end of your manuscript, and update any in-text citations to match accordingly. Please see our Supporting Information guidelines for more information: http://journals.plos.org/plosone/s/supporting-information.](http://track.editorialmanager.com/CL0/http:%2F%2Fjournals.plos.org%2Fplosone%2Fs%2Fsupporting-information./1/010f019edda2304d-3e0d82a5-fc1c-4439-8ee2-4be1e6a7209a-000000/E4WeKZ20tL1GecQlEf_dFR20SP0Dw3R0S8hMralfYSA=258) | Thank you, this has been rectified. | 382 |
| In addition to addressing all reviewer comments, please revise the manuscript as follows:   1. Remove the subheading “Study Limitations” currently appearing at line 302. Incorporate the limitations into the final paragraph of the Discussion section instead. 2. Add future directions based on the key findings of the study in the last paragraph of the Conclusion. 3. Update the reference list to ensure that at least 80% of citations are from the past five years | These have been addressed accordingly. | 330, 346-348 |
| **Reviewer 1** |  |  |
| 1. The claim that "no previous study had published the prevalence of adequate Fruit and Vegetable consumption among PLHIV" is inaccurate and requires revision. Relevant prior estimates exist in comparable Sub-Saharan African populations, including Boneya et al. (2024), who reported that 76.34% of HIV infected adults in Ethiopia consumed Fruits and Vegetables less than once per day, and Mosha et al. (2026), who found that only 25% of HIV negative adults and 38% of PLHIV consumed fruits four or more days per week in Tanzania. These estimates could and should have informed a more evidence-based prevalence assumption rather than defaulting to 50%. | We have revised the discussion including this current literature. | 247-262 |
| 2. Given the observed prevalence of 10.6%, the authors should justify their choice of modified Poisson regression over logistic regression, ideally supported by a sensitivity analysis comparing both approaches. | Thank you, we selected the modified Poisson regression as the prevalence was slightly above 10%, although logistic regression could provide similar estimates, odds ratios may still overestimate the magnitude of association. | NA |
| 3. No internal consistency testing such as Cronbach's alpha is reported for the Health Belief Model constructs. The cutoff of 3 or above for "favourable" perceptions and the collapsing of "neutral" responses into the "unfavourable" category are both arbitrary decisions that require explicit justification. | We appreciate this observation. Cronbach’s alpha was not assessed in the original analysis and has now been acknowledged as a limitation. The HBM constructs were adapted from previously validated measures, and the categorization approach was based on the direction of responses rather than a validated threshold. We have added this as a limitation. | 333-339 |
| 4. No approach to handling missing data is described, particularly for clinically abstracted variables such as viral load, which may be missing for some patients. | Thank you for this observation. We have added a description of our approach to handling missing data in the Methods section. For clinically abstracted variables, including viral load, analyses were conducted using available data, and participants with missing values for specific variables were excluded from analyses involving those variables only. We did not perform data imputation because the proportion of missing data was low. | 185-187 |
| **Reviewer 2** |  |  |
| Style and Format For Layout and spacing, it is required that Manuscript text should be double-spaced. For references, In the text, cite the reference number in square brackets (e.g., “We used the techniques developed by our colleagues [19] to analyze the data”). The PLOS One Manuscript Organization section states that figure files should not be included in the manuscript body but rather should be uploaded separately. In addition, the figure captions should be placed in the main manuscript text immediately after the paragraph in which the figure is first cited. | Thank you, this has been done. | NA |
| Line 212: Remove ‘a’ from “that eating a fruits and vegetables” | Thank you, this has been revised. | 212 |
| Line 213-214: “However, most participants perceived the high cost (47.3%, 204/431) and limited variety (42.0%, 181/431) of fruits and vegetables at eateries as major barriers to their consumption”. Did the authors assess how frequently participants eat at eateries? Without this information, it is difficult to determine whether these factors meaningfully influence overall fruit and vegetable consumption. In addition, was there any association between employment status and the perception that eateries offer limited or expensive fruit and vegetable options? | Thank you for this comment. We did not collect data on how frequently participants ate meals at eateries; therefore, we are unable to determine the extent to which these perceived barriers influenced their overall fruit and vegetable consumption. We also did not examine the association between employment status and perceptions regarding the cost or availability of fruits and vegetables at eateries. To avoid overinterpretation, we have revised the text to emphasize that these findings reflect participants' perceptions rather than established determinants of fruit and vegetable intake. | 540-544 |
| Check Table 2: The row for “Overall perceptions about fruits and vegetables Consumption” has a mean value but no frequency data. Table 3: Adjust the column heading to align with the corresponding data | Thank you for this observation. The "Overall perceptions about fruits and vegetables consumption" variable represents a composite score calculated by averaging responses across multiple perception items measured on a Likert scale. As such, it does not have a corresponding single agree/disagree frequency. We have revised the table by indicating that frequency data are not applicable for this composite measure and clarified this in the table footnote.  Table 3 columns have been aligned accordingly. | 540-544 |
| Line 262: Adjust the reference style to fit PLOS guideline References: It is recommended that the authors should update the references to include recent references to reflect current knowledge in this field. | Thank you, this has been rectified. | 262 |
