# Supplementary material for: Fruit and vegetable consumption among people living with HIV at a large Urban HIV Clinic in Central Uganda: A cross-sectional study
Source: PLoS One. 2026 Nov 6;21(11):e0361755. doi: 10.1371/journal.pone.0361755 (PMC13742917; doi:10.1371/journal.pone.0361755)
Supplement: Supplementary file 2 [file pone.0361755.s004.docx]

Response to Reviewers’ letter

Manuscript title: “Fruit and vegetable consumption among people living with HIV at a large Urban HIV Clinic in Central Uganda: a cross-sectional study.”

Thank you for reviewing our manuscript. Below is a point-by-point response to each comment.

Reviewer #1:

Comment

Thank you for revising most of my comments. I appreciate the changes made. However, my first comment in the Sample Size Estimation section still needs clarification.

The statement that “no previous study had published the prevalence of adequate fruit and vegetable consumption among PLHIV” does not appear to be accurate. Previous studies have reported fruit and vegetable intake among PLHIV, including Sousa (2019), Boneya et al. (2024), and Mosha et al. (2026). Boneya et al. (2024), for example, specifically assessed fruit and vegetable intake among adults receiving ART in Ethiopia.

Relevant links:
Sousa (2019): <https://doi.org/10.31080/ASNH.2019.03.0548>
Boneya et al. (2024): <https://www.frontiersin.org/journals/nutrition/articles/10.3389/fnut.2024.1380987/full>
Mosha et al. (2026): <https://pubmed.ncbi.nlm.nih.gov/41683251/>

Please consider revising this statement and providing a clearer justification for using 50% in the sample size calculation. If 50% was used as a conservative estimate because no sufficiently comparable prevalence estimate was available at the time of study planning, this would be a more appropriate explanation.

Response:

Thank you for your comment. We have revised the statement to read “The sample size was calculated using Kish and Leslie formula (22) based on the following assumptions: a 5% level of significance, assumed prevalence of 50% for fruit and vegetable consumption (because no sufficiently comparable prevalence estimate was available at the time of study planning), an error margin of 5% and a 10% non-response rate. This gave us a minimum sample size of 427 participants.”
