# Supplementary material for: Fruit and vegetable consumption among people living with HIV at a large Urban HIV Clinic in Central Uganda: A cross-sectional study
Source: PLoS One. 2026 Nov 6;21(11):e0361755. doi: 10.1371/journal.pone.0361755 (PMC13742917; doi:10.1371/journal.pone.0361755)
Supplement: Peer review history

# Peer review: Decision Letter 0

**DOI:** 10.1371/journal.pone.0361755.r001

**Authors:** Mabel Aworh

18 Jun 2026

Dear Dr. Nantale,

Thank you for submitting your manuscript to PLOS ONE. After careful consideration, we feel that it has merit but does not fully meet PLOS ONE’s publication criteria as it currently stands. Therefore, we invite you to submit a revised version of the manuscript that addresses the points raised during the review process.

Please submit your revised manuscript by Aug 02 2026 11:59PM. If you will need more time than this to complete your revisions, please reply to this message or contact the journal office at plosone@plos.org. When you're ready to submit your revision, log on to https://www.editorialmanager.com/pone/ and select the 'Submissions Needing Revision' folder to locate your manuscript file.

- A letter that responds to each point raised by the academic editor and reviewer(s). You should upload this letter as a separate file labeled 'Response to Reviewers'.
- A marked-up copy of your manuscript that highlights changes made to the original version. You should upload this as a separate file labeled 'Revised Manuscript with Track Changes'.
- An unmarked version of your revised paper without tracked changes. You should upload this as a separate file labeled 'Manuscript'.

If you would like to make changes to your financial disclosure, please include your updated statement in your cover letter. Guidelines for resubmitting your figure files are available below the reviewer comments at the end of this letter.

If applicable, we recommend that you deposit your laboratory protocols in protocols.io to enhance the reproducibility of your results. Protocols.io assigns your protocol its own identifier (DOI) so that it can be cited independently in the future. For instructions see: https://journals.plos.org/plosone/s/submission-guidelines#loc-laboratory-protocols. Additionally, PLOS ONE offers an option for publishing peer-reviewed Lab Protocol articles, which describe protocols hosted on protocols.io. Read more information on sharing protocols at https://plos.org/protocols?utm_medium=editorial-email&utm_source=authorletters&utm_campaign=protocols.

As the corresponding author, your ORCID iD is verified in the submission system and will appear in the published article. PLOS supports the use of ORCID, and we encourage all coauthors to register for an ORCID iD and use it as well. Please encourage your coauthors to verify their ORCID iD within the submission system before final acceptance, as unverified ORCID iDs will not appear in the published article. *Only*  the individual author can complete the verification step; PLOS staff *cannot*  verify ORCID iDs on behalf of authors.

We look forward to receiving your revised manuscript.

Kind regards,

Mabel Kamweli Aworh, DVM, MPH, PhD. FCVSN

Academic Editor

PLOS One

Journal Requirements:

When submitting your revision, we need you to address these additional requirements.

1. Please ensure that your manuscript meets PLOS ONE's style requirements, including those for file naming. The PLOS ONE style templates can be found at

https://journals.plos.org/plosone/s/file?id=wjVg/PLOSOne_formatting_sample_main_body.pdf and https://journals.plos.org/plosone/s/file?id=ba62/PLOSOne_formatting_sample_title_authors_affiliations.pdf

2. We note that you had received approval to conduct research from Uganda National Council of Science and Technology (UNCST) number HS3597ES. If this is the case, please add this information to your manuscript and specify that this institution granted permission to conduct research.

3. Please include your full ethics statement in the ‘Methods’ section of your manuscript file. In your statement, please include the full name of the IRB or ethics committee who approved or waived your study, as well as whether or not you obtained informed written or verbal consent. If consent was waived for your study, please include this information in your statement as well.

4. Please provide a complete Data Availability Statement in the submission form, ensuring you include all necessary access information or a reason for why you are unable to make your data freely accessible. If your research concerns only data provided within your submission, please write "All data are in the manuscript and/or supporting information files" as your Data Availability Statement.

5. Please amend your list of authors on the manuscript to ensure that each author is linked to an affiliation. Authors’ affiliations should reflect the institution where the work was done (if authors moved subsequently, you can also list the new affiliation stating “current affiliation:….” as necessary).

6. We note you have included a table to which you do not refer in the text of your manuscript. Please ensure that you refer to Table 1, 2, 3, in your text; if accepted, production will need this reference to link the reader to the Table.

7. Please include captions for your Supporting Information files at the end of your manuscript, and update any in-text citations to match accordingly. Please see our Supporting Information guidelines for more information: http://journals.plos.org/plosone/s/supporting-information.

8. We are unable to open your Supporting Information file [dataset pub mart.dta]. Please kindly revise as necessary and re-upload.

9. If the reviewer comments include a recommendation to cite specific previously published works, please review and evaluate these publications to determine whether they are relevant and should be cited. There is no requirement to cite these works unless the editor has indicated otherwise.

Additional Editor Comments :

In addition to addressing all reviewer comments, please revise the manuscript as follows:

1. Remove the subheading “Study Limitations” currently appearing at line 302. Incorporate the limitations into the final paragraph of the Discussion section instead.
2. Add future directions based on the key findings of the study in the last paragraph of the Conclusion.
3. Update the reference list to ensure that at least 80% of citations are from the past five years.

[Note: HTML markup is below. Please do not edit.]

Reviewers' comments:

Reviewer's Responses to Questions

**Comments to the Author**

1. Is the manuscript technically sound, and do the data support the conclusions?

Reviewer #1: Partly

Reviewer #2: Yes

**********

2. Has the statistical analysis been performed appropriately and rigorously?

Reviewer #1: No

Reviewer #2: Yes

**********

3. Have the authors made all data underlying the findings in their manuscript fully available?

The PLOS Data policy

Reviewer #1: Yes

Reviewer #2: Yes

**********

4. Is the manuscript presented in an intelligible fashion and written in standard English?

Reviewer #1: Yes

Reviewer #2: Yes

**********

Reviewer #1: Thank you for submitting this manuscript to the journal. The study addresses an important and underexplored topic regarding fruit and vegetable consumption among people living with HIV in Uganda, and the work is appreciated. However, several concerns require attention before the manuscript can be considered for publication. They are as follows:

1. The claim that "no previous study had published the prevalence of adequate Fruit and Vegetable consumption among PLHIV" is inaccurate and requires revision. Relevant prior estimates exist in comparable Sub-Saharan African populations, including Boneya et al. (2024), who reported that 76.34% of HIV infected adults in Ethiopia consumed Fruits and Vegetables less than once per day, and Mosha et al. (2026), who found that only 25% of HIV negative adults and 38% of PLHIV consumed fruits four or more days per week in Tanzania. These estimates could and should have informed a more evidence-based prevalence assumption rather than defaulting to 50%.

2. Given the observed prevalence of 10.6%, the authors should justify their choice of modified Poisson regression over logistic regression, ideally supported by a sensitivity analysis comparing both approaches.

3. No internal consistency testing such as Cronbach's alpha is reported for the Health Belief Model constructs. The cutoff of 3 or above for "favourable" perceptions and the collapsing of "neutral" responses into the "unfavourable" category are both arbitrary decisions that require explicit justification.

4. No approach to handling missing data is described, particularly for clinically abstracted variables such as viral load, which may be missing for some patients.

Reviewer #2: This manuscript investigates the frequency of fruit and vegetable consumption and the factors influencing their intake among people living with HIV. Overall, the study is well organized and contributes useful information to existing literature. However, some aspects of the manuscript would benefit from further clarification and correction. The comments and suggestions below are intended to strengthen the report and improve the interpretation of the findings.

Style and Format

For Layout and spacing, it is required that Manuscript text should be double-spaced.

For references, In the text, cite the reference number in square brackets (e.g., “We used the techniques developed by our colleagues [19] to analyze the data”).

The PLOS One Manuscript Organization section states that figure files should not be included in the manuscript body but rather should be uploaded separately. In addition, the figure captions should be placed in the main manuscript text immediately after the paragraph in which the figure is first cited.

Line 212: Remove ‘a’ from “that eating a fruits and vegetables”

Line 213-214: “However, most participants perceived the high cost (47.3%, 204/431) and limited variety (42.0%, 181/431) of fruits and vegetables at eateries as major barriers to their consumption”. Did the authors assess how frequently participants eat at eateries? Without this information, it is difficult to determine whether these factors meaningfully influence overall fruit and vegetable consumption. In addition, was there any association between employment status and the perception that eateries offer limited or expensive fruit and vegetable options?

Check Table 2: The row for “Overall perceptions about fruits and vegetables

Consumption” has a mean value but no frequency data.

Table 3: Adjust the column heading to align with the corresponding data

Line 262: Adjust the reference style to fit PLOS guideline

References: It is recommended that the authors should update the references to include recent references to reflect current knowledge in this field.

**********

what does this mean?). If published, this will include your full peer review and any attached files.

If you choose “no”, your identity will remain anonymous but your review may still be made public.

**Do you want your identity to be public for this peer review?**  For information about this choice, including consent withdrawal, please see our Privacy Policy

Reviewer #1: No

Reviewer #2: **Yes:**  Damilola Odumade

**********

[NOTE: If reviewer comments were submitted as an attachment file, they will be attached to this email and accessible via the submission site. Please log into your account, locate the manuscript record, and check for the action link "View Attachments". If this link does not appear, there are no attachment files.]

To ensure your figures meet our technical requirements, please review our figure guidelines: https://journals.plos.org/plosone/s/figures

You may also use PLOS’s free figure tool, NAAS, to help you prepare publication quality figures: https://journals.plos.org/plosone/s/figures#loc-tools-for-figure-preparation.

NAAS will assess whether your figures meet our technical requirements by comparing each figure against our figure specifications.

# Author comment: Author response to Decision Letter 1

**DOI:** 10.1371/journal.pone.0361755.r002

18 Aug 2026

Re: “PONE-D-26-17920

Fruit and vegetable consumption among people living with HIV at a large Urban HIV Clinic in Central Uganda: a cross-sectional study”

Thank you for reviewing our manuscript.

Below is our point-by-point response to each comment.

Thank you.

Comment Response to comment Line number

Editor

1. Please ensure that your manuscript meets PLOS ONE's style requirements, including those for file naming.

Thank you, this has been done. NA

2. We note that you had received approval to conduct research from Uganda National Council of Science and Technology (UNCST) number HS3597ES. If this is the case, please add this information to your manuscript and specify that this institution granted permission to conduct research. We received from approval from Makerere University School of Public Health Research and Ethical Committee, approval (number MakSPH-REC-2023-229) and we highlighted this in the manuscript under Ethics approval and consent to participate. 354

3. Please include your full ethics statement in the ‘Methods’ section of your manuscript file. In your statement, please include the full name of the IRB or ethics committee who approved or waived your study, as well as whether or not you obtained informed written or verbal consent. If consent was waived for your study, please include this information in your statement as well. Thank you, this has been done. 354-361

4. Please provide a complete Data Availability Statement in the submission form, ensuring you include all necessary access information or a reason for why you are unable to make your data freely accessible. If your research concerns only data provided within your submission, please write "All data are in the manuscript and/or supporting information files" as your Data Availability Statement. Thank you, this has been done. 368

5. Please amend your list of authors on the manuscript to ensure that each author is linked to an affiliation. Authors’ affiliations should reflect the institution where the work was done (if authors moved subsequently, you can also list the new affiliation stating “current affiliation:….” as necessary). Thank you, each author is linked to an affiliation. 4-5

6. We note you have included a table to which you do not refer in the text of your manuscript. Please ensure that you refer to Table 1, 2, 3, in your text; if accepted, production will need this reference to link the reader to the Table. Thank you, this has been done. 199,226,236

7. Please include captions for your Supporting Information files at the end of your manuscript, and update any in-text citations to match accordingly. Please see our Supporting Information guidelines for more information: http://journals.plos.org/plosone/s/supporting-information.

Thank you, this has been rectified. 382

In addition to addressing all reviewer comments, please revise the manuscript as follows:

1. Remove the subheading “Study Limitations” currently appearing at line 302. Incorporate the limitations into the final paragraph of the Discussion section instead.

2. Add future directions based on the key findings of the study in the last paragraph of the Conclusion.

3. Update the reference list to ensure that at least 80% of citations are from the past five years

These have been addressed accordingly. 330, 346-348

Reviewer 1

1. The claim that "no previous study had published the prevalence of adequate Fruit and Vegetable consumption among PLHIV" is inaccurate and requires revision. Relevant prior estimates exist in comparable Sub-Saharan African populations, including Boneya et al. (2024), who reported that 76.34% of HIV infected adults in Ethiopia consumed Fruits and Vegetables less than once per day, and Mosha et al. (2026), who found that only 25% of HIV negative adults and 38% of PLHIV consumed fruits four or more days per week in Tanzania. These estimates could and should have informed a more evidence-based prevalence assumption rather than defaulting to 50%. We have revised the discussion including this current literature. 247-262

2. Given the observed prevalence of 10.6%, the authors should justify their choice of modified Poisson regression over logistic regression, ideally supported by a sensitivity analysis comparing both approaches. Thank you, we selected the modified Poisson regression as the prevalence was slightly above 10%, although logistic regression could provide similar estimates, odds ratios may still overestimate the magnitude of association. NA

3. No internal consistency testing such as Cronbach's alpha is reported for the Health Belief Model constructs. The cutoff of 3 or above for "favourable" perceptions and the collapsing of "neutral" responses into the "unfavourable" category are both arbitrary decisions that require explicit justification. We appreciate this observation. Cronbach’s alpha was not assessed in the original analysis and has now been acknowledged as a limitation. The HBM constructs were adapted from previously validated measures, and the categorization approach was based on the direction of responses rather than a validated threshold. We have added this as a limitation. 333-339

4. No approach to handling missing data is described, particularly for clinically abstracted variables such as viral load, which may be missing for some patients. Thank you for this observation. We have added a description of our approach to handling missing data in the Methods section. For clinically abstracted variables, including viral load, analyses were conducted using available data, and participants with missing values for specific variables were excluded from analyses involving those variables only. We did not perform data imputation because the proportion of missing data was low. 185-187

Reviewer 2

Style and Format

For Layout and spacing, it is required that Manuscript text should be double-spaced.

For references, In the text, cite the reference number in square brackets (e.g., “We used the techniques developed by our colleagues [19] to analyze the data”).

The PLOS One Manuscript Organization section states that figure files should not be included in the manuscript body but rather should be uploaded separately. In addition, the figure captions should be placed in the main manuscript text immediately after the paragraph in which the figure is first cited. Thank you, this has been done. NA

Line 212: Remove ‘a’ from “that eating a fruits and vegetables”

Thank you, this has been revised.

212

Line 213-214: “However, most participants perceived the high cost (47.3%, 204/431) and limited variety (42.0%, 181/431) of fruits and vegetables at eateries as major barriers to their consumption”. Did the authors assess how frequently participants eat at eateries? Without this information, it is difficult to determine whether these factors meaningfully influence overall fruit and vegetable consumption. In addition, was there any association between employment status and the perception that eateries offer limited or expensive fruit and vegetable options? Thank you for this comment. We did not collect data on how frequently participants ate meals at eateries; therefore, we are unable to determine the extent to which these perceived barriers influenced their overall fruit and vegetable consumption. We also did not examine the association between employment status and perceptions regarding the cost or availability of fruits and vegetables at eateries. To avoid overinterpretation, we have revised the text to emphasize that these findings reflect participants' perceptions rather than established determinants of fruit and vegetable intake. 540-544

Check Table 2: The row for “Overall perceptions about fruits and vegetables

Consumption” has a mean value but no frequency data.

Table 3: Adjust the column heading to align with the corresponding data

Thank you for this observation. The "Overall perceptions about fruits and vegetables consumption" variable represents a composite score calculated by averaging responses across multiple perception items measured on a Likert scale. As such, it does not have a corresponding single agree/disagree frequency. We have revised the table by indicating that frequency data are not applicable for this composite measure and clarified this in the table footnote.

Table 3 columns have been aligned accordingly. 540-544

Line 262: Adjust the reference style to fit PLOS guideline

References: It is recommended that the authors should update the references to include recent references to reflect current knowledge in this field. Thank you, this has been rectified. 262

Attachment Submitted filename: Fruit and Vegetables paper_response to reviewers_.docx

# Peer review: Decision Letter 1

**DOI:** 10.1371/journal.pone.0361755.r003

**Authors:** Mabel Aworh

7 Oct 2026

Dear Dr. Nantale,

Thank you for submitting your manuscript to PLOS One. After careful consideration, we feel that it has merit but does not fully meet PLOS One’s publication criteria as it currently stands. Therefore, we invite you to submit a revised version of the manuscript that addresses the points raised during the review process.

Please submit your revised manuscript by Nov 21 2026 11:59PM. If you will need more time than this to complete your revisions, please reply to this message or contact the journal office at plosone@plos.org. When you're ready to submit your revision, log on to https://www.editorialmanager.com/pone/ and select the 'Submissions Needing Revision' folder to locate your manuscript file.

- A letter that responds to each point raised by the academic editor and reviewer(s). You should upload this letter as a separate file labeled 'Response to Reviewers'.
- A marked-up copy of your manuscript that highlights changes made to the original version. You should upload this as a separate file labeled 'Revised Manuscript with Track Changes'.
- An unmarked version of your revised paper without tracked changes. You should upload this as a separate file labeled 'Manuscript'.

If you would like to make changes to your financial disclosure, please include your updated statement in your cover letter. Guidelines for resubmitting your figure files are available below the reviewer comments at the end of this letter.

If applicable, we recommend that you deposit your laboratory protocols in protocols.io to enhance the reproducibility of your results. Protocols.io assigns your protocol its own identifier (DOI) so that it can be cited independently in the future. For instructions see: https://journals.plos.org/plosone/s/submission-guidelines#loc-laboratory-protocols. Additionally, PLOS One offers an option for publishing peer-reviewed Lab Protocol articles, which describe protocols hosted on protocols.io. Read more information on sharing protocols at https://plos.org/protocols?utm_medium=editorial-email&utm_source=authorletters&utm_campaign=protocols.

As the corresponding author, your ORCID iD is verified in the submission system and will appear in the published article. PLOS supports the use of ORCID, and we encourage all coauthors to register for an ORCID iD and use it as well. Please encourage your coauthors to verify their ORCID iD within the submission system before final acceptance, as unverified ORCID iDs will not appear in the published article. *Only*  the individual author can complete the verification step; PLOS staff *cannot*  verify ORCID iDs on behalf of authors.

We look forward to receiving your revised manuscript.

Kind regards,

Mabel Kamweli Aworh, DVM, MPH, PhD. FCVSN

Academic Editor

PLOS One

Journal Requirements:

If the reviewer comments include a recommendation to cite specific previously published works, please review and evaluate these publications to determine whether they are relevant and should be cited. There is no requirement to cite these works unless the editor has indicated otherwise.

Please review your reference list to ensure that it is complete and correct. If you have cited papers that have been retracted, please include the rationale for doing so in the manuscript text, or remove these references and replace them with relevant current references. Any changes to the reference list should be mentioned in the rebuttal letter that accompanies your revised manuscript. If you need to cite a retracted article, indicate the article’s retracted status in the References list and also include a citation and full reference for the retraction notice.

[Note: HTML markup is below. Please do not edit.]

Reviewers' comments:

Reviewer's Responses to Questions

**Comments to the Author**

Reviewer #1: (No Response)

Reviewer #2: All comments have been addressed

**********

2. Is the manuscript technically sound, and do the data support the conclusions?

Reviewer #1: Yes

Reviewer #2: Yes

**********

3. Has the statistical analysis been performed appropriately and rigorously?

Reviewer #1: Yes

Reviewer #2: N/A

**********

4. Have the authors made all data underlying the findings in their manuscript fully available?

The PLOS Data policy

Reviewer #1: Yes

Reviewer #2: Yes

**********

5. Is the manuscript presented in an intelligible fashion and written in standard English?

Reviewer #1: Yes

Reviewer #2: Yes

**********

Reviewer #1: Thank you for revising most of my comments. I appreciate the changes made. However, my first comment in the Sample Size Estimation section still needs clarification.

The statement that “no previous study had published the prevalence of adequate fruit and vegetable consumption among PLHIV” does not appear to be accurate. Previous studies have reported fruit and vegetable intake among PLHIV, including Sousa (2019), Boneya et al. (2024), and Mosha et al. (2026). Boneya et al. (2024), for example, specifically assessed fruit and vegetable intake among adults receiving ART in Ethiopia.

Relevant links:

Sousa (2019): https://doi.org/10.31080/ASNH.2019.03.0548

Boneya et al. (2024): https://www.frontiersin.org/journals/nutrition/articles/10.3389/fnut.2024.1380987/full

Mosha et al. (2026): https://pubmed.ncbi.nlm.nih.gov/41683251/

Please consider revising this statement and providing a clearer justification for using 50% in the sample size calculation. If 50% was used as a conservative estimate because no sufficiently comparable prevalence estimate was available at the time of study planning, this would be a more appropriate explanation.

Reviewer #2: (No Response)

**********

what does this mean?). If published, this will include your full peer review and any attached files.

If you choose “no”, your identity will remain anonymous but your review may still be made public.

**Do you want your identity to be public for this peer review?**  For information about this choice, including consent withdrawal, please see our Privacy Policy

Reviewer #1: No

Reviewer #2: No

**********

[NOTE: If reviewer comments were submitted as an attachment file, they will be attached to this email and accessible via the submission site. Please log into your account, locate the manuscript record, and check for the action link "View Attachments". If this link does not appear, there are no attachment files.]

To ensure your figures meet our technical requirements, please review our figure guidelines: https://journals.plos.org/plosone/s/figures

You may also use PLOS’s free figure tool, NAAS, to help you prepare publication quality figures: https://journals.plos.org/plosone/s/figures#loc-tools-for-figure-preparation.

NAAS will assess whether your figures meet our technical requirements by comparing each figure against our figure specifications.

# Author comment: Author response to Decision Letter 2

**DOI:** 10.1371/journal.pone.0361755.r004

9 Oct 2026

Response to Reviewers’ letter

Manuscript title: “Fruit and vegetable consumption among people living with HIV at a large Urban HIV Clinic in Central Uganda: a cross-sectional study.”

Thank you for reviewing our manuscript. Below is a point-by-point response to each comment.

Reviewer #1:

Comment

Thank you for revising most of my comments. I appreciate the changes made. However, my first comment in the Sample Size Estimation section still needs clarification.

The statement that “no previous study had published the prevalence of adequate fruit and vegetable consumption among PLHIV” does not appear to be accurate. Previous studies have reported fruit and vegetable intake among PLHIV, including Sousa (2019), Boneya et al. (2024), and Mosha et al. (2026). Boneya et al. (2024), for example, specifically assessed fruit and vegetable intake among adults receiving ART in Ethiopia.

Relevant links:

Sousa (2019): https://doi.org/10.31080/ASNH.2019.03.0548

Boneya et al. (2024): https://www.frontiersin.org/journals/nutrition/articles/10.3389/fnut.2024.1380987/full

Mosha et al. (2026): https://pubmed.ncbi.nlm.nih.gov/41683251/

Please consider revising this statement and providing a clearer justification for using 50% in the sample size calculation. If 50% was used as a conservative estimate because no sufficiently comparable prevalence estimate was available at the time of study planning, this would be a more appropriate explanation.

Response:

Thank you for your comment. We have revised the statement to read “The sample size was calculated using Kish and Leslie formula (22) based on the following assumptions: a 5% level of significance, assumed prevalence of 50% for fruit and vegetable consumption (because no sufficiently comparable prevalence estimate was available at the time of study planning), an error margin of 5% and a 10% non-response rate. This gave us a minimum sample size of 427 participants.”

Attachment Submitted filename: Response to Reviewers 081026.docx

# Editor report: Decision Letter 2

**DOI:** 10.1371/journal.pone.0361755.r005

**Authors:** Mabel Aworh

16 Oct 2026

Fruit and vegetable consumption among people living with HIV at a large Urban HIV Clinic in Central Uganda: a cross-sectional study

PONE-D-26-17920R2

Dear Dr. Nantale,

We’re pleased to inform you that your manuscript has been judged scientifically suitable for publication and will be formally accepted for publication once it meets all outstanding technical requirements.

Within one week, you’ll receive an e-mail detailing the required amendments. When these have been addressed, you’ll receive a formal acceptance letter and your manuscript will be scheduled for publication.

An invoice will be generated when your article is formally accepted. Please note, if your institution has a publishing partnership with PLOS and your article meets the relevant criteria, all or part of your publication costs will be covered. Please make sure your user information is up-to-date by logging into Editorial Manager at Editorial Manager® and clicking the ‘Update My Information' link at the top of the page. For questions related to billing, please contact billing support.

If your institution or institutions have a press office, please notify them about your upcoming paper to help maximize its impact. If they’ll be preparing press materials, please inform our press team as soon as possible -- no later than 48 hours after receiving the formal acceptance. Your manuscript will remain under strict press embargo until 2 pm Eastern Time on the date of publication. For more information, please contact onepress@plos.org.

Kind regards,

Mabel Kamweli Aworh, DVM, MPH, PhD. FCVSN

Academic Editor

PLOS One

Additional Editor Comments (optional):

Reviewers' comments:

# Editor report: Acceptance letter

**DOI:** 10.1371/journal.pone.0361755.r006

**Authors:** Mabel Aworh

PONE-D-26-17920R2

PLOS One

Dear Dr. Nantale,

I'm pleased to inform you that your manuscript has been deemed suitable for publication in PLOS One. Congratulations! Your manuscript is now being handed over to our production team.

At this stage, our production department will prepare your paper for publication. This includes ensuring the following:

* All references, tables, and figures are properly cited

* All relevant supporting information is included in the manuscript submission,

* There are no issues that prevent the paper from being properly typeset

You will receive further instructions from the production team, including instructions on how to review your proof when it is ready. Please keep in mind that we are working through a large volume of accepted articles, so please give us a few days to review your paper and let you know the next and final steps.

Lastly, if your institution or institutions have a press office, please let them know about your upcoming paper now to help maximize its impact. If they'll be preparing press materials, please inform our press team within the next 48 hours. Your manuscript will remain under strict press embargo until 2 pm Eastern Time on the date of publication. For more information, please contact onepress@plos.org.

You will receive an invoice from PLOS for your publication fee after your manuscript has reached the completed accept phase. If you receive an email requesting payment before acceptance or for any other service, this may be a phishing scheme. Learn how to identify phishing emails and protect your accounts at https://explore.plos.org/phishing.

If we can help with anything else, please email us at customercare@plos.org.

Thank you for submitting your work to PLOS One and supporting open access.

Kind regards,

PLOS One Editorial Office Staff

on behalf of

Dr. Mabel Kamweli Aworh

Academic Editor

PLOS One
